# Supplementary material for: scAPAmod: Profiling Alternative Polyadenylation Modalities in Single Cells from Single-Cell RNA-Seq Data
Source: Int J Mol Sci. 2022 Jul 23;23(15):8123. doi: 10.3390/ijms23158123 (PMC9329739; doi:10.3390/ijms23158123)
Supplement: Supplementary file 1 [file ijms-23-08123-s001.zip › 20220701-scAPAmod Supp Figures.pdf]

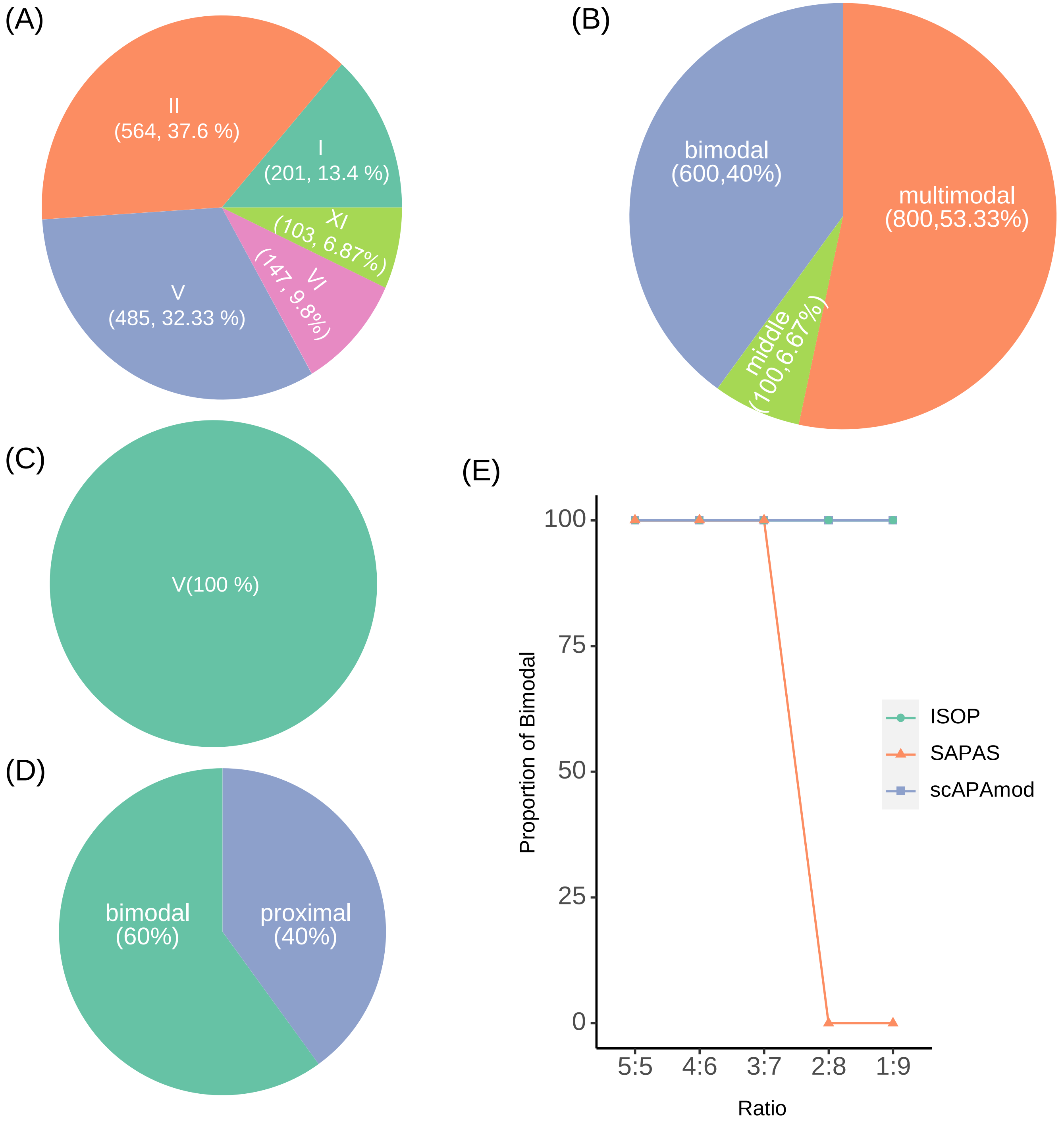

Figure S1. Simulation results of ISOP and SAPAS on the 100-cell simulation data. (A) The results of ISOP from noise-free simulation data; (B) The results of SAPAS from noise-free simulation data; (C) For simulation data with five different cell numbers ratios for the two components of the bimodality, ISOP identifies all as V pattern; (D) For simulation data with five different cell numbers ratios for the two components of the bimodality, 60% of the identification results of SAPAS is bimodal pattern and 40% is proximal pattern; (E) The results of scAPAmod, ISOP and SAPAS from the data of the bimodal pattern with varied cell numbers ratio of the two components.

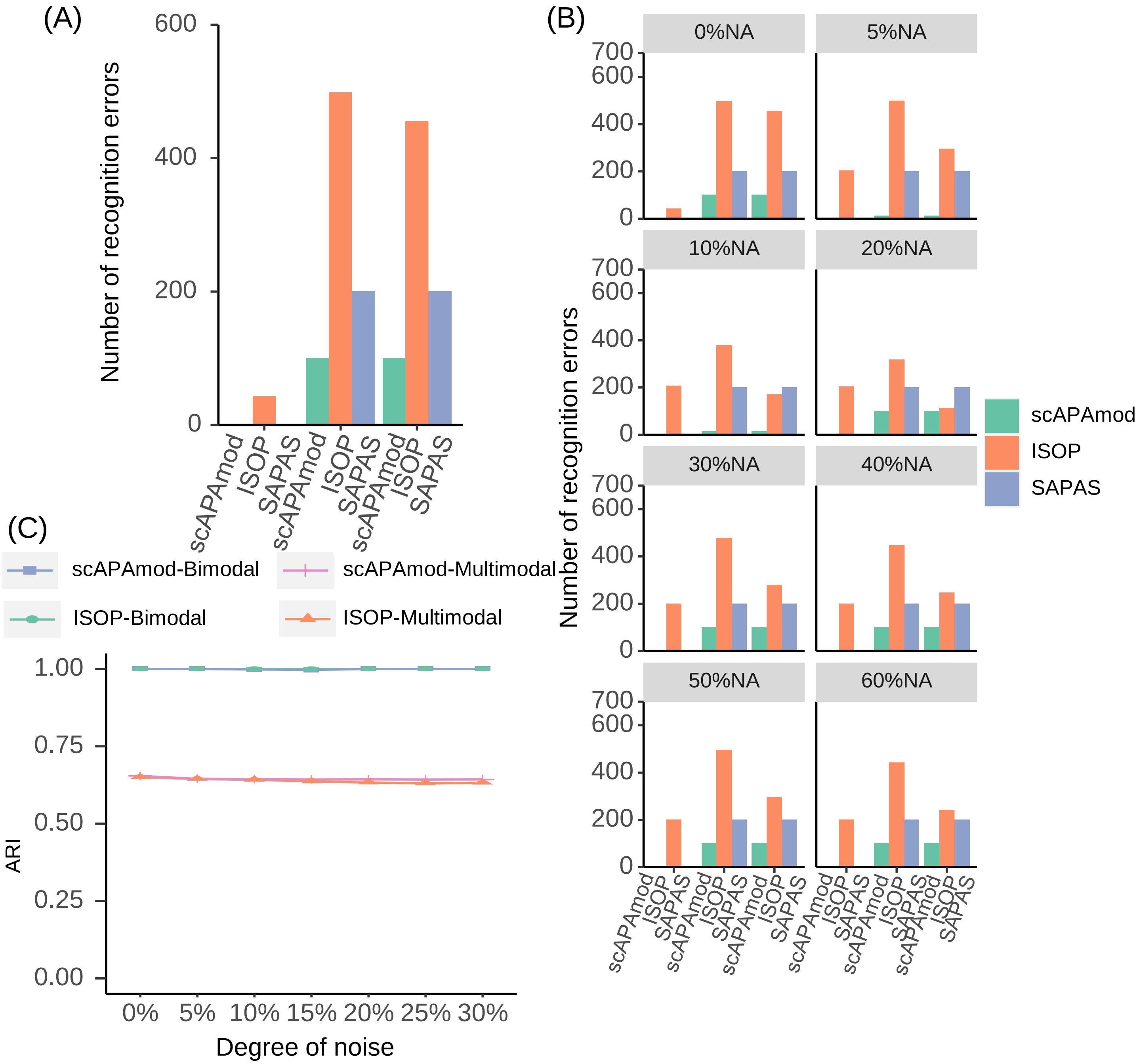

Figure S2. Simulation results of the 8000-cell simulation data. (A) Number of recognition errors of different modalities from noise-free simulated data. (B) Number of recognition errors of different modalities from the simulated data with different degrees of missing values. (C) ARI of simulated data with different degrees of noise.

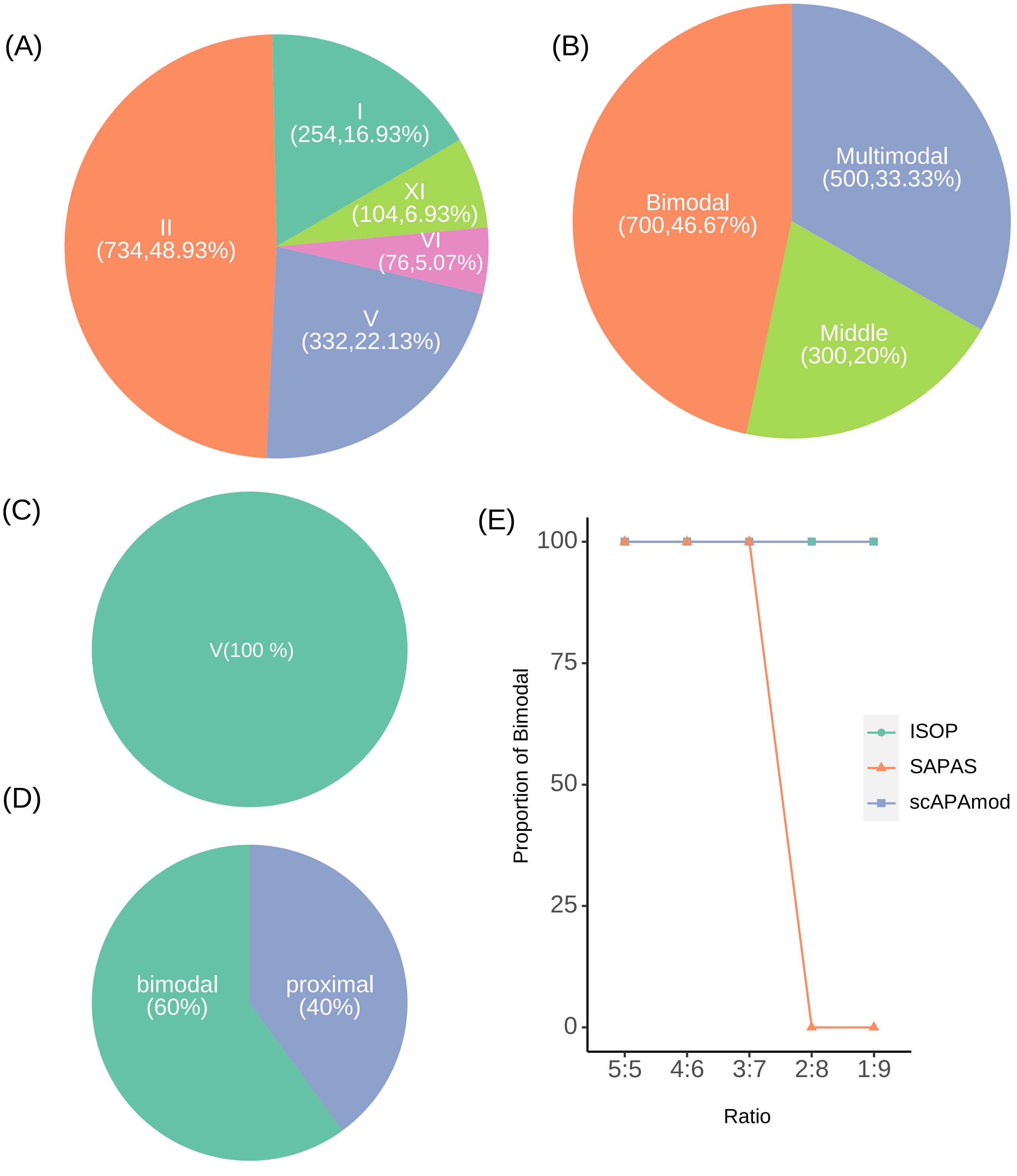

Figure S3. Simulation results of ISOP and SAPAS on the 8000-cell simulation data. (A) The results of ISOP from noise-free simulation data; (B) The results of SAPAS from noise-free simulation data; (C) For simulation data with five different cell ratios for the two components of the bimodality, ISOP identifies all as V pattern; (D) For simulation data with five different cell ratios for the two components of the bimodality, 60% of the identification results of SAPAS is bimodal pattern and 40% is proximal pattern; (E) The results of scAPAmod, ISOP and SAPAS from the data of the bimodal pattern with varied cell numbers ratio of the two components.

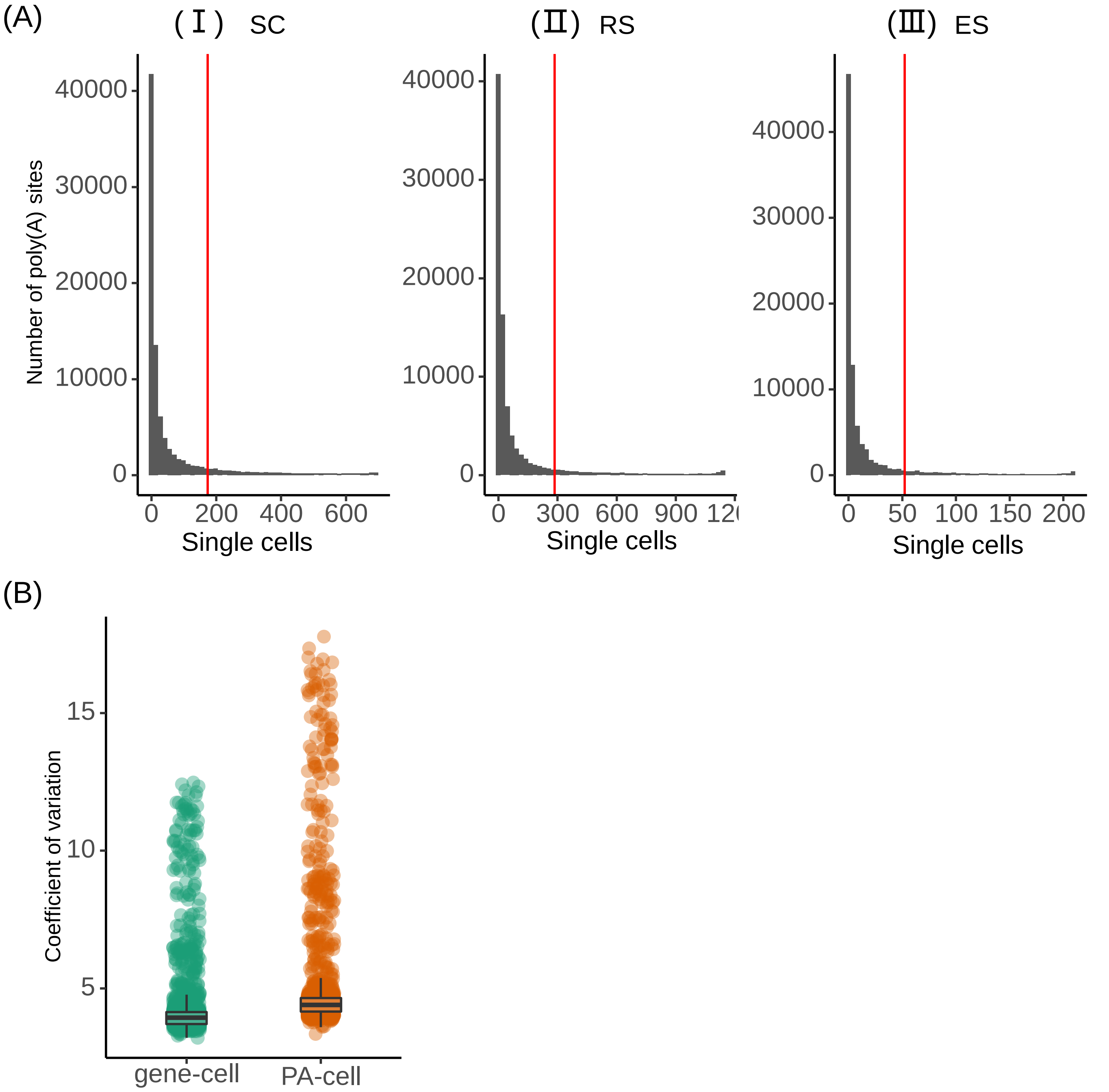

Figure S4. The expression of poly(A) sites in mouse spermatogenesis cells. (A) The distributions of the number of poly(A) sites expressed in mouse spermatogenesis cells. the horizontal axis represents the number of cells with expression in poly(A) sites, the vertical axis represents the number of corresponding poly(A) sites, and the red line represents the expression of these poly(A) sites in 1/4 of total cells; (B) The coefficient of variation of expression of gene and poly(A) sites of the 2042 cells.

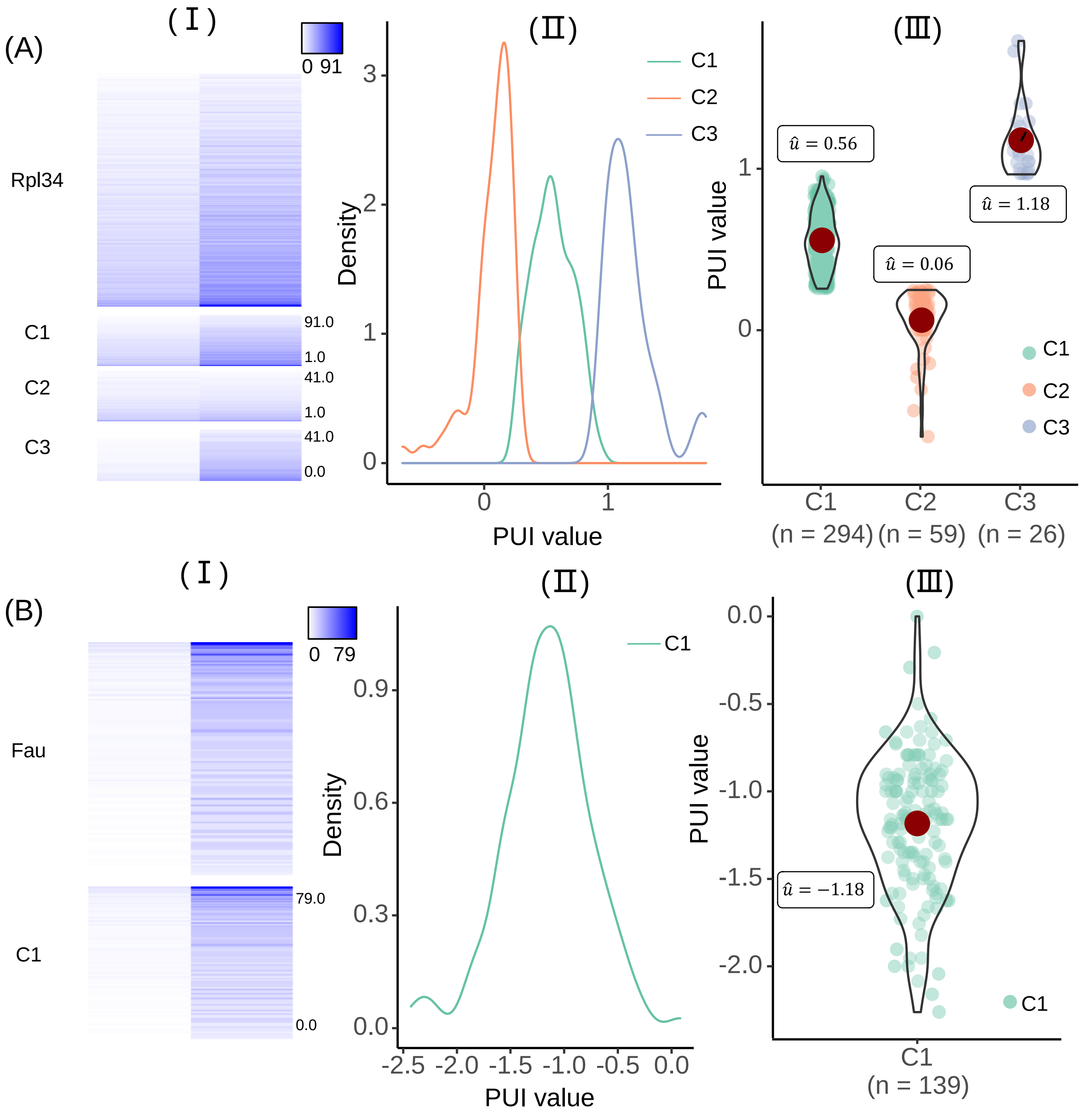

Figure S5. Usage patterns of gene Rpl34 and Fau. (A) The distribution of the poly(A) sites expression (I), the density curve of PUI value (II), and the distribution of PUI data (III) of gene Rpl34; (B) gene Fau is identified as unimodal, and the picture shows similar (A).

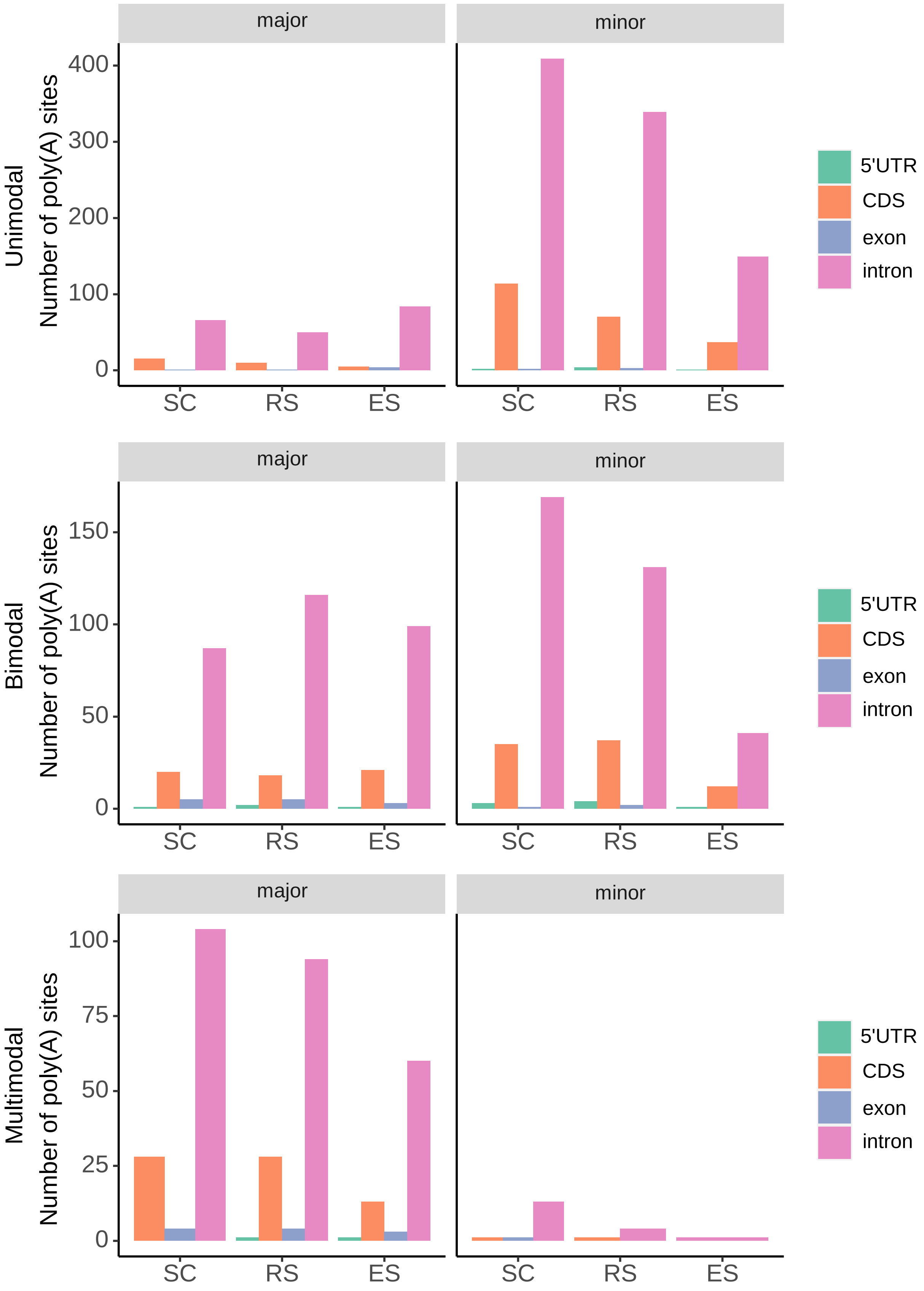

Figure S6. Statistics on the number of major poly(A) sites and minor poly(A) sites detectable usage patterns of non-3' UTR. Some genes contain exons. Poly(A) sites found in such genes are located in exons. Poly(A) sites found in protein coding genes are located in CDS and UTR.

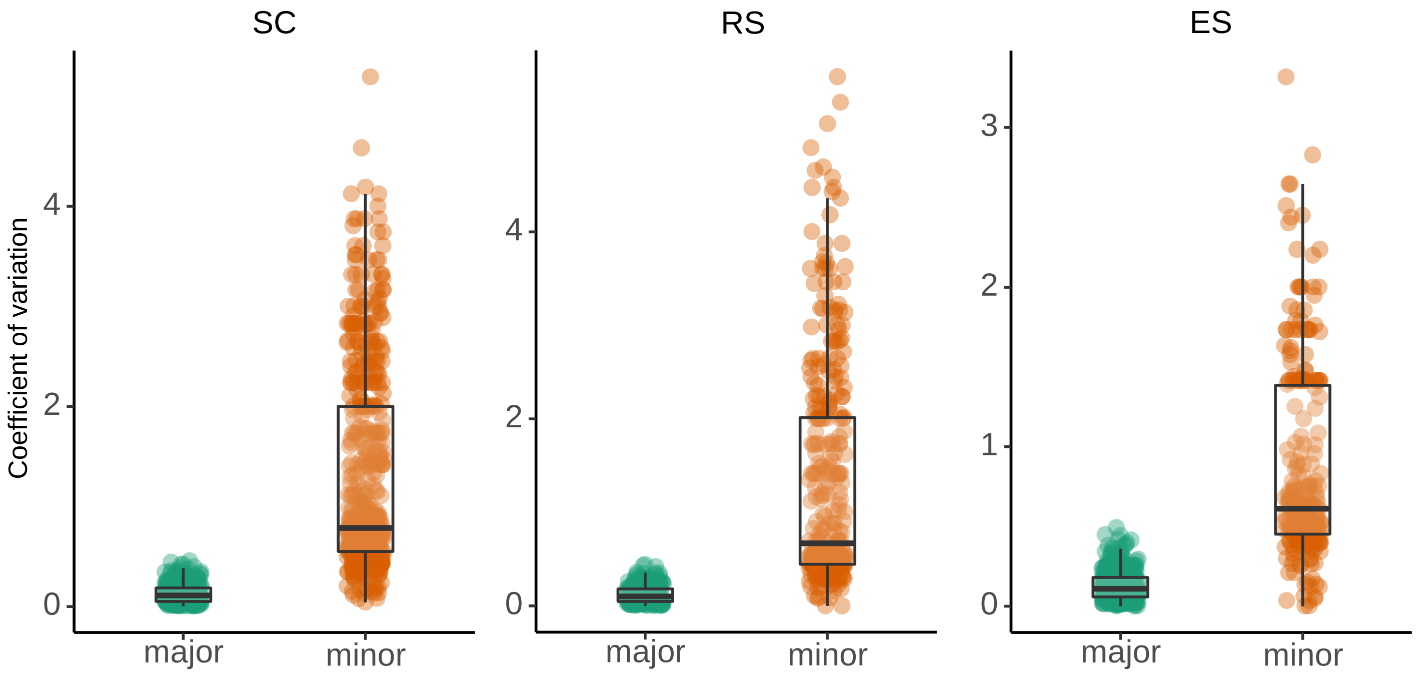

Figure S7. the coefficient of variation of usages of major and minor poly(A) sites in three cell types based on expression levels

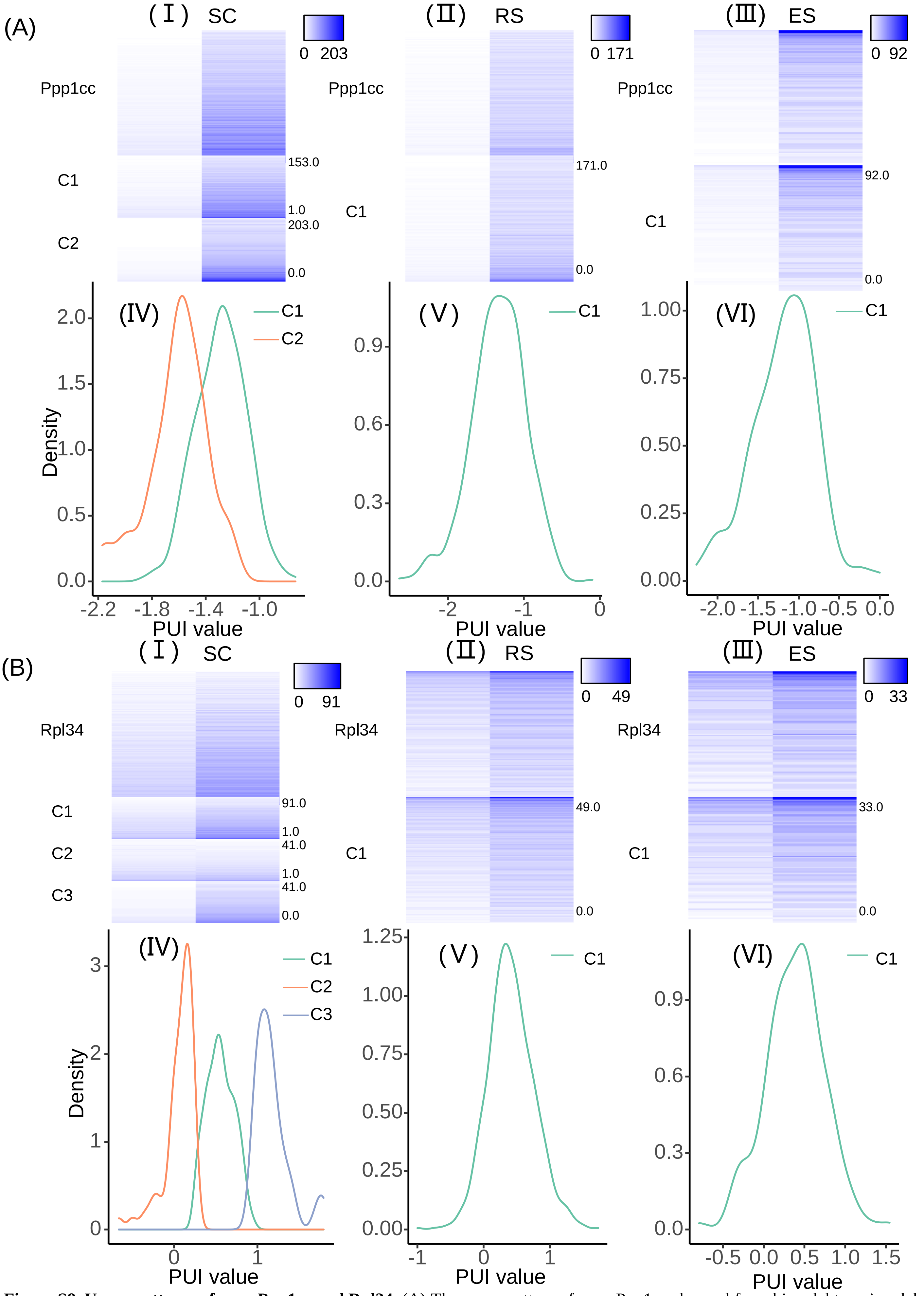

Figure S8. Usage patterns of gene Ppp1cc and Rpl34. (A) The usage pattern of gene Ppp1cc changed from bimodal to unimodal in different cell types; the distribution of the two poly(A) sites expression (upper), the density curve of PUI value (lower) of gene Ppp1cc. (B) The usage pattern of gene Rpl34 changed from multimodal to unimodal in different cell types.

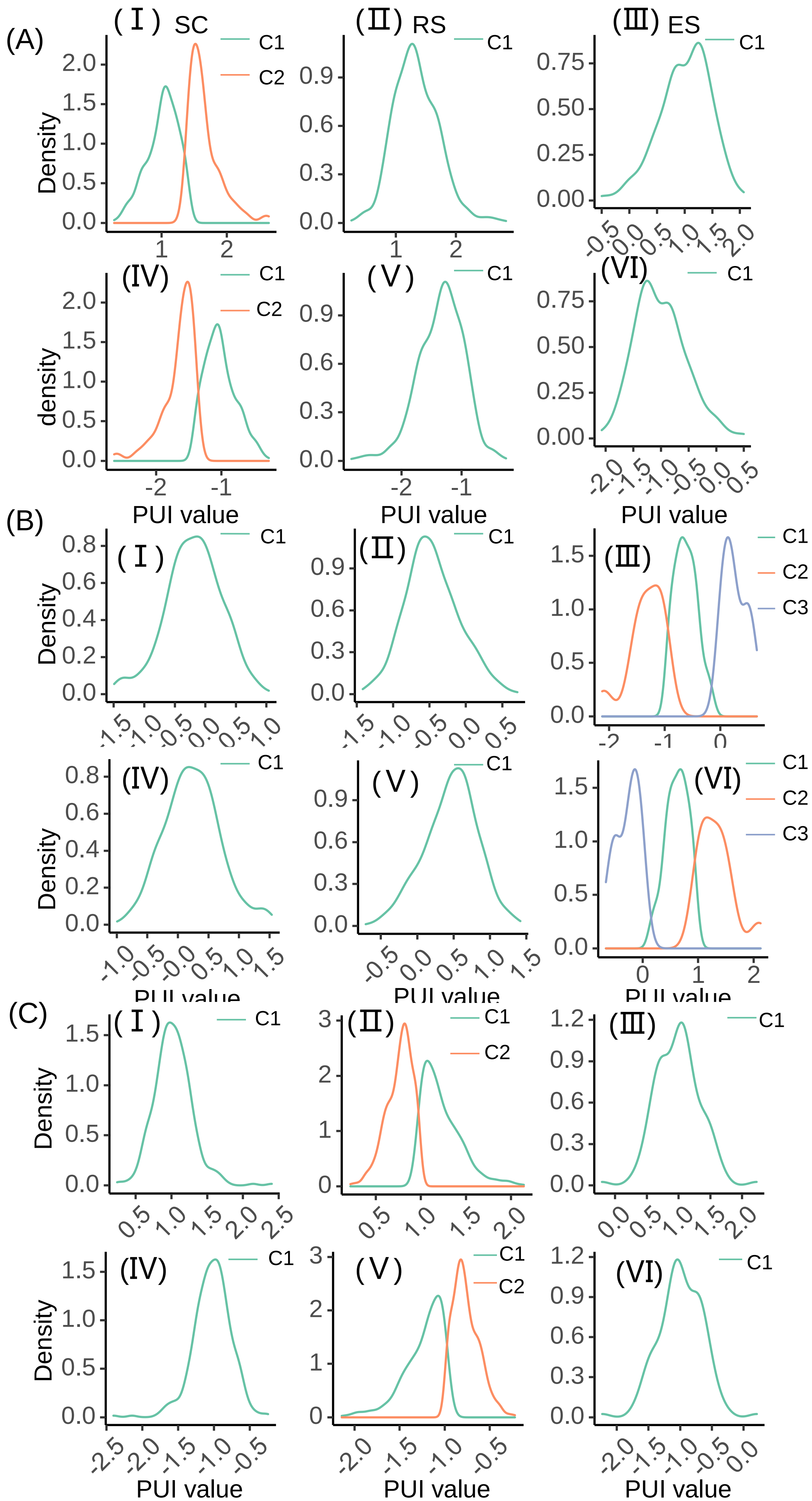

Figure S9. Usage patterns changes of the gene Ddx5, Pabpc1 and Cetn1 in non-3' UTR. (A) The usage pattern of gene Ddx5 changed from bimodal to unimodal across the three cell types; (B) The usage pattern of gene Pabpc1 changed from unimodal to multimodal across the three cell types; (C) The usage pattern of gene Cetn1 changed from unimodal to bimodal, and then to unimodal across the three cell types.

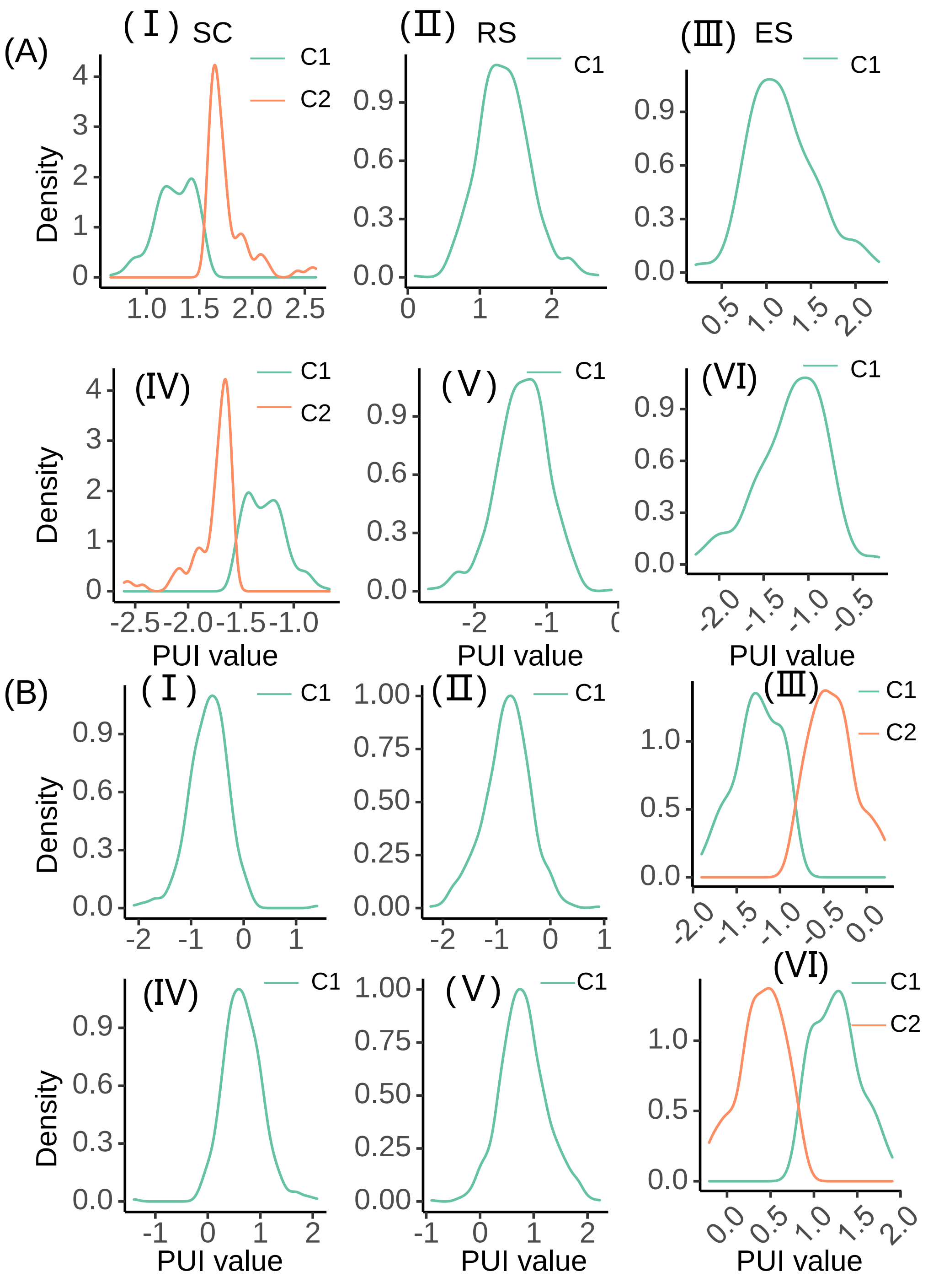

Figure S10. Usage patterns changes of the gene Ppp1cc and Srpk1 in non-3’ UTR region. (A) The usage pattern of gene Ppp1cc changed from bimodal to unimodal across the three cell types; (B) The usage pattern of gene Srpk1 changed from unimodal to bimodal across the three cell types.

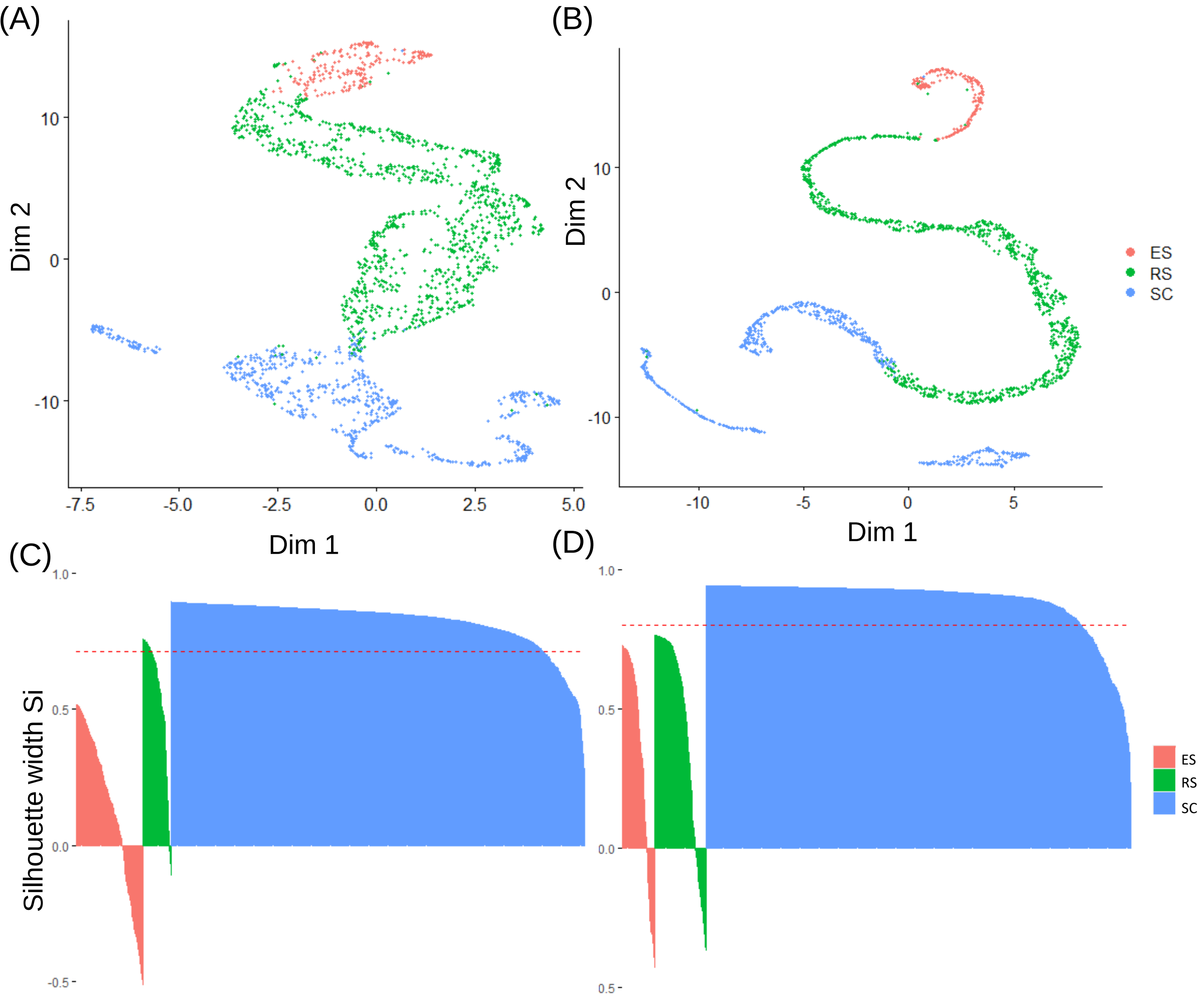

Figure S11. (A, B) Scatter plots showing UMAP results of 2042 cells based on the APA expression profile of genes with APA modalities identified by scAPAmod (A), the gene expression profile of all genes (B). Cells are colored and labelled according to the cell type annotations from the real labels. (C, D) Silhouette plots for clustering results of 2042 cells based on the APA expression profile of genes with APA modalities identified by scAPAmod (C), the gene expression profile of all genes (D). The x-axis represents cells, and y-axis is the corresponding silhouette coefficient Si for each cell. The silhouette coefficient measures how similar a cell is to its own cluster compared with other clusters. The red dashed line is the average Si for all cells.
